# Reduced erythrocyte deformability associated with hypoargininemia during *Plasmodium* *falciparum* malaria

**DOI:** 10.1038/srep03767

**Published:** 2014-01-20

**Authors:** Juliana Rey, Pierre A. Buffet, Liliane Ciceron, Geneviève Milon, Odile Mercereau-Puijalon, Innocent Safeukui

**Affiliations:** 1 Institut Pasteur, Immunologie Moléculaire des Parasites, Département de Parasitologie Mycologie, F- 75015 Paris, France; 2 CNRS, URA2581, F- 75015 Paris, France; 3 INSERM - UPMC (Paris 6 University) UMRs945, F-75013 Paris, France; 4 AP-HP, Department of Parasitology, Pitié Salpétrière Hospital, F-75013 Paris, France; 5 Institut Pasteur, Immunophysiologie et Parasitisme, Département de Parasitologie Mycologie, F-75015 Paris, France; 6 Center for Rare and Neglected Diseases, and Department of Biological Sciences, University of Notre Dame, Notre Dame, Indiana, USA

## Abstract

The mechanisms underlying reduced red blood cell (RBC) deformability during *Plasmodium falciparum* (*Pf*) malaria remain poorly understood. Here, we explore the possible involvement of the L-arginine and nitric oxide (NO) pathway on RBC deformability in *Pf*-infected patients and parasite cultures. RBC deformability was reduced during the acute attack (day0) and returned to normal values upon convalescence (day28). Day0 values correlated with plasma L-arginine levels (r = 0.69; p = 0.01) and weakly with parasitemia (r = −0.38; p = 0.006). *In vitro*, day0 patient's plasma incubated with ring-stage cultures at 41°C reduced RBC deformability, and this effect correlated strongly with plasma L-arginine levels (r = 0.89; p < 0.0001). Moreover, addition of exogenous L-arginine to the cultures increased deformability of both *Pf*-free and trophozoite-harboring RBCs. NO synthase activity, evidenced in *Pf*-infected RBCs, induced L-arginine-dependent NO production. These data show that hypoargininemia during *P.* *falciparum* malaria may altogether impair NO production and reduce RBC deformability, particularly at febrile temperature.

The reduced deformability of the red blood cell (RBC) contributes to the pathogenesis of several hematological disorders^1^,^2^ and *Plasmodium* *falciparum* malaria^3^,^4^,^5^ by impairing RBC circulation through systemic capillaries^6^ and splenic slits^2^,^7^,^8^,^9^. The reduced deformability of RBC harboring *P. falciparum* (*Pf*RBC)^7^,^8^,^9^,^10^,^11^ and their splenic entrapment^7^,^8^,^9^ are well documented. However, little is known about the reduced deformability of uninfected RBCs (uRBCs) during malaria and their handling by the spleen, a phenomenon that may also impair microcirculatory flow^12^, and possibly trigger their retention in the spleen, thereby contributing to malarial anemia.

Ecktacytometry measurements showed that whole peripheral RBCs were less deformable during acute *P. falciparum* malaria compared to healthy subjects^3^,^4^. This was exacerbated in patients with severe infection, particularly in children with severe malarial anemia, and associated with a poor prognosis^3^,^4^. Reduced RBC deformability in malaria has been attributed to increased rigidity of *Pf*RBCs and, quite importantly, of uRBCs as well^3^,^4^.

Several factors may drive the reduced deformability of uRBCs during malaria, including rigidifying substances released upon schizont rupture, uptake by the membranes of uRBCs^13^,^14^, deposition of parasite molecules on uRBCs during abortive invasion^15^, or malaria-induced variations in the concentration of systemic host factors such as nitric oxide (NO)^16^,^17^,^18^. Indeed, NO has been shown to improve animal^19^,^20^ and human^21^,^22^,^23^ RBC deformability.

NO is produced by nitric oxide synthase (NOS) which catalyzes the conversion of L-arginine to L-citrulline. Human RBCs possess an active endothelial NOS^23^, and incubation of RBCs with L-arginine increases their deformability, an effect inhibited by specific inhibitors of mammalian NOS^21^,^22^,^23^. RBC NOS shares the common substrate L-arginine with arginase I, which is present in high concentration in RBCs and catalyses the hydrolysis of L-arginine to L-ornithine and urea. Arginase may participate in the regulation of NO production during malaria by reducing the bioavailability of L-arginine^24^,^25^,^26^,^27^.

The role of NO during *P. falciparum* infection has been intensively investigated^16^,^28^,^29^, and there is now good evidence for impairment of NO bioavailability in uncomplicated and particularly severe malaria in African children^28^,^29^. The molecular basis of this impairement is not fully clear, although a possible mechanism could be consumption of the arginine precursor due to the high parasite arginase activity^26^,^27^, which has been shown to be the major determinant of L-arginine depletion in *P. falciparum* cultures^26^. Indeed, reduced plasma L-arginine levels correlate with decreased NO production^18^, and have been associated with severe malaria and death^18^,^30^. However, to our knowledge, there is no study documenting the effect of NO on the mechanical properties of RBCs during malaria.

The work reported here aims at clarifying the role of L-arginine and NO pathway on the deformability of RBCs during *P.* *falciparum* malaria. We analyzed the relationship between L-arginine and nitrite levels, parasitemia and RBC deformability in patients with acute uncomplicated *P. falciparum* malaria, and explored the effect of patient plasma on the deformability of *P. falciparum* ring-stage cultures *in vitro*.

## Results

### Reduced red blood cell deformability during acute malaria attack correlates with decreased plasma concentrations of L-arginine

**Figure 1:**
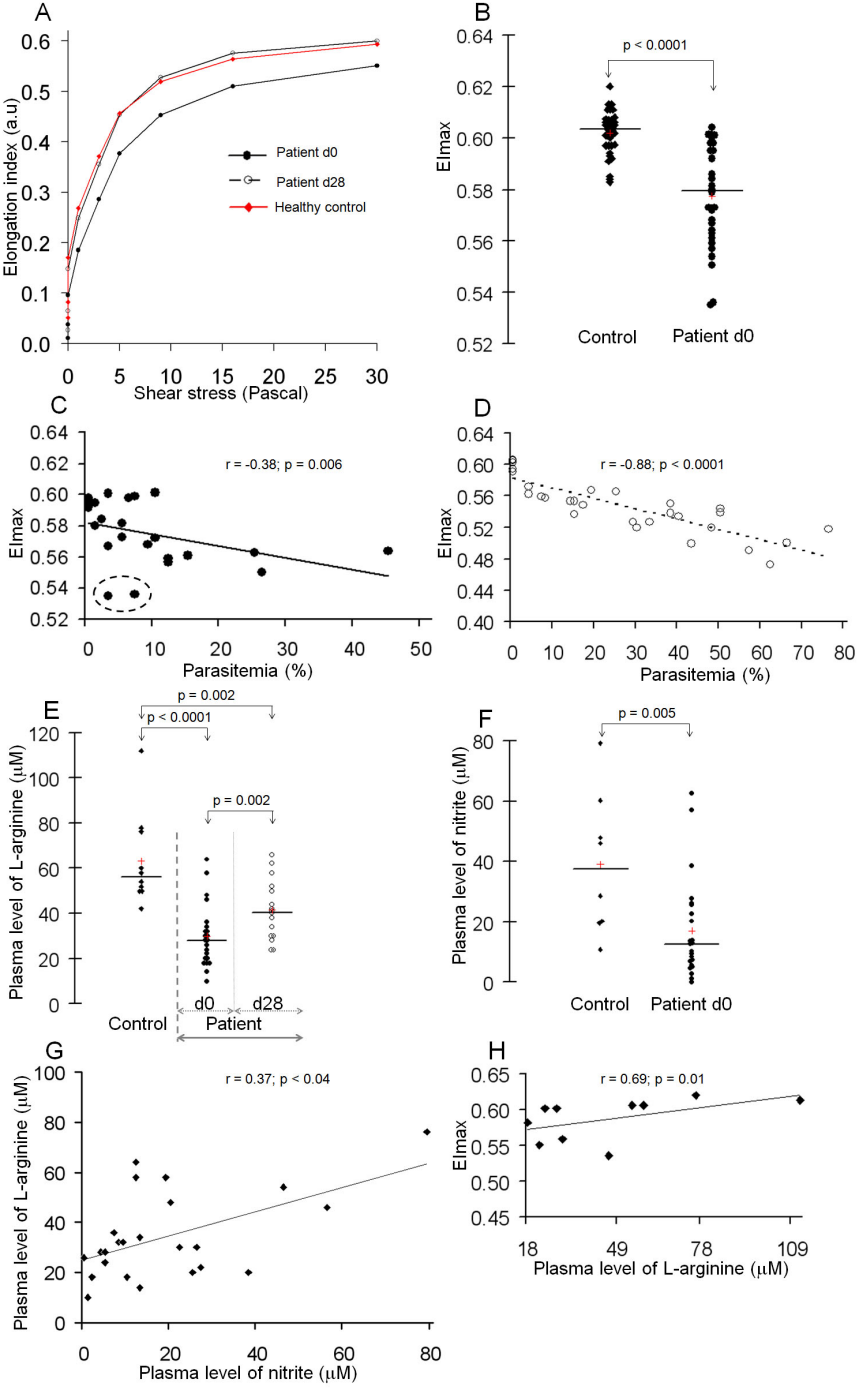
Correlation between plasma concentrations of L-arginine and deformability of whole peripheral red blood cells from malaria patients. A representative deformability profile of whole peripheral RBCs from a *P.* *falciparum* malaria patient at day0 (d0; parasitemia: 25%) during acute attack and 28 days after clinical recovery (d28) compared to a healthy control ((A), see also Supplementary Figure 1 for the all total 30 patients) and maximal EI (EImax or EI at 30 Pascal of shear stress) of whole peripheral RBCs from the 30 malaria patients during clinical symptoms at day0 (d0) compared to healthy controls (B). Blood samples from blood bank (Etablissement Français du Sang, Paris) from 30 healthy subjects who have never travelled to malaria endemic areas were used as a control group. Linear regression fit of the correlation between EImax values of whole RBCs and parasite densities of either malaria patients at d0 (C) or cultured synchronous ring stages (D). Distribution of plasma levels of L-arginine of patients at day0 (d0, n = 22) and day28 (d28, n = 16) (E), and of nitrite of patients at day0 (d0, n = 22) (F). Plasmas obtained from healthy Europeans subjects who have never travelled to endemic areas were used as controls (n = 10). Linear regression fit of the correlation between plasma levels of L-arginine and nitrite of malaria patients at day0 (G). Linear regression fit of the correlation between EImax and the plasma levels of L-arginine of malaria patients at day0 (H).

We evaluated the deformability of whole blood from 30 individuals with *P. falciparum* uncomplicated malaria at admission (day0). The clinical and biological characteristics of the malaria patients are summarized in Table 1. Blood samples from 30 healthy blood bank donors who had never travelled to malaria-endemic areas were used as a control group. Ektacytometry analysis showed reduced deformability of the patients' whole RBCs compared to healthy controls (Figure 1A for a representative patient, Supplementary Figure 1 for all 30 patients). Elongation index (EI, a deformability parameter) values of patient RBCs at day0 were lower compared to the control group (Figure 1B), and returned to normal values 28 days after clinical recovery (day28) (Figure 1A) suggesting that the lower EI values of patients at day0 were linked to malaria.

RBC deformability of patients estimated by the Ecktacytometer at day0 is a summation of the cellular deformability of both uninfected (uRBCs) and infected (*Pf*RBCs) RBCs fractions, with a contribution to the overall values proportional to their respective size^31^. Since the vast majority of RBCs in malaria patients are uRBCs, the reduction in RBC deformability results mainly from changes in the uRBCs. The modest contribution of the infected RBC fraction to this measure was supported by the weak correlation between parasitemia at day0 and maximal EI values (EI max or EI at a shear stress of 30 Pascal) (r = −0.38; p = 0.006) (Figure 1C), contrasting with the strong correlation between EI max and parasitemia of cultured synchronous ring-stages (r = −0.88; p < 0.0001) (Figure 1D). Measurements in two individuals with very low parasitemia (that cannot be detected with Ecktacytometer if *Pf*RBCs exclusively contribute to the reduced deformability of whole RBCs) displayed the lowest EI max values. (Figure 1C; circled). Together, these results suggest that parasitemia *per se* (the fraction of *Pf*RBCs in the sample) is not the sole explanatory variable for the reduced EI values of the whole RBC population of patients during malaria episodes and that uRBCs may also be involved, confirming conclusions of studies with patients living in endemic regions^3^,^4^.

We then measured the concentrations of L-arginine and nitrite in cryopreserved plasma from malaria patients at day0 and day28. Cryopreserved plasma from healthy Europeans blood donors who had never travelled to endemic areas were used as controls. L-arginine plasma levels were lower in patients at day0 (mean ± sd: 29.8 ± 13.8 μmol/L) compared to day28 (41.4 ± 13.2 μmol/L, p = 0.002) or healthy controls (63.2 ± 20.6 μmol/L, p < 0.0001) (Figure 1E). The plasma concentrations of nitrite were lower in malaria patients at day0 (17.1 ± 16.6 μmol/L) compared to healthy controls (39.0 ± 23.4 μmol/L, p = 0.005) (Figure 1F). At day0, the plasma level of L-arginine positively correlated with the concentration of nitrite (r = 0.37; p < 0.04) (Figure 1G). There was a strong and positive correlation between the plasma concentrations of L-arginine and the EI max values of patient RBCs at day0 (r = 0.69; p = 0.01) (Figure 1H). No correlation was observed between EI max and the level of nitrite at day0 (r = −0.06; p = 0.87).

### Reduced deformability of cultured ring-stages induced by incubation with day0 patient plasma correlated with plasma concentration of L-arginine

**Figure 2:**
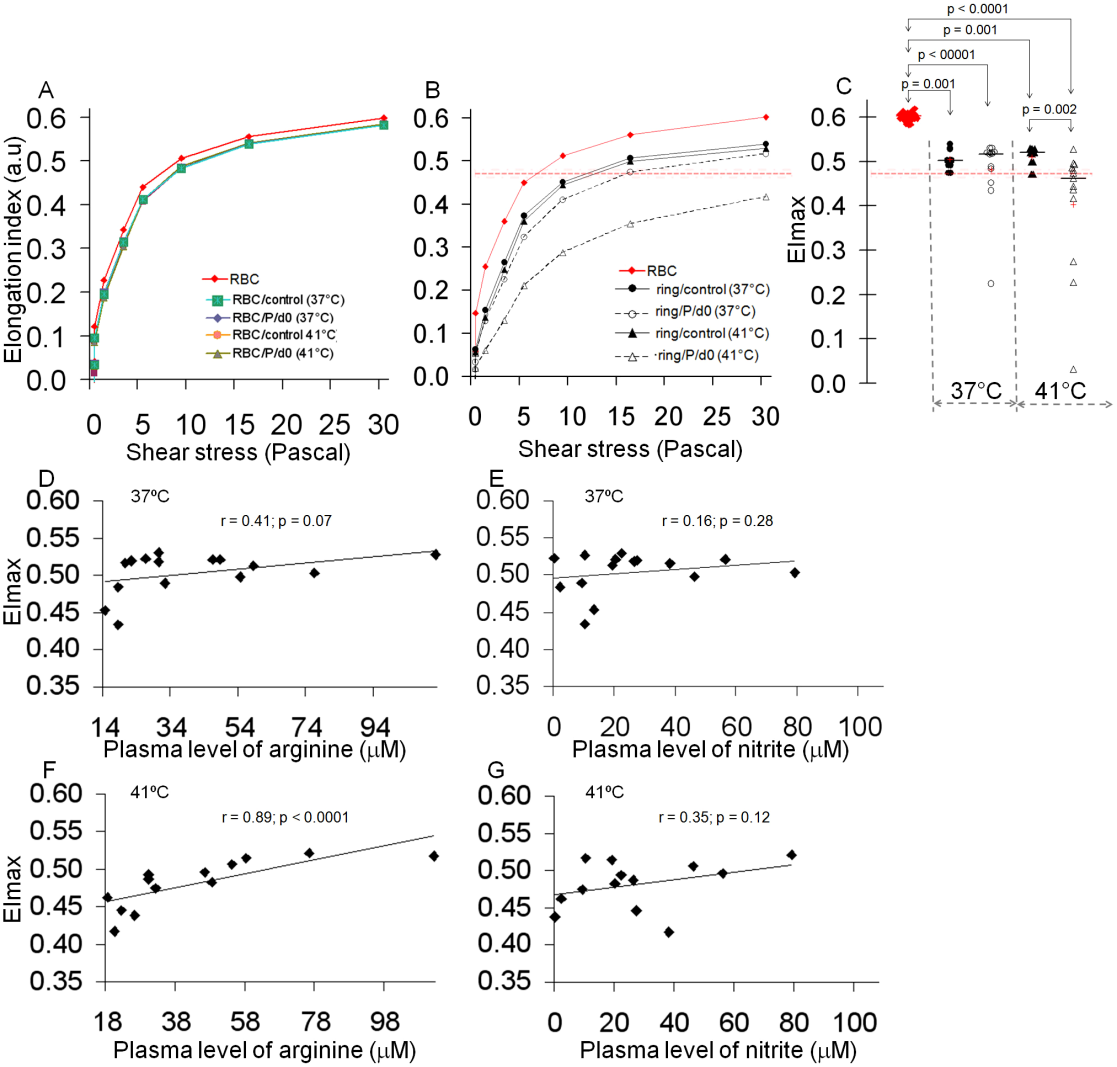
Increased rigidity of *P. falciparum* ring-stage cultures incubated with plasma of malaria patients collected during acute attack correlates with plasma levels of L-arginine. The deformability profile of healthy RBCs (A) or a ring-stage culture (B) incubated at 37 or 41°C as indicated with plasma (P) collected at day0 (d0) from a malaria patient (P) or with control plasma (control). EImax of ring-stage cultures incubated at 37 or 41°C with malaria patient's plasma from day0 (d0) (n = 12) or with control plasma (n = 8) (C). The median (interquantile range) of EImax values of cultured rings (parasitemia: 43–63%) incubated at 41°C with patient's plasmas coincide with the estimates for 100% ring-stage harboring RBCs preparations in a previous study^7^. The pink band and the middle line (red color) on figures 2B–C represent, respectively, the interquartile range and median of the estimated EImax values for 100% ring harboring RBCs parasitemia. Linear regression fit of the correlation between EImax of a ring-stage culture incubated at 37°C with patients' plasmas and the plasma levels of L-arginine (D) or nitrite (E). Linear regression fit of the correlation between EImax of ring culture incubated at 41°C with patients' plasmas and the plasma levels of L-arginine (F) or nitrite (G).

We next investigated whether plasma collected from malaria patients at day0 could alter cellular deformability of cultured ring-stages. We incubated cultured ring-stages (parasitemia: 43 – 63% depending on the preparation) for 2 hours at 37 or 41°C with the cryopreserved plasma samples collected at day0 from patients with uncomplicated *P. falciparum* malaria, and measured their EI by Ektacytometry (Supplementary Figure 2A–L). Eight plasmas from age-matched malaria-naive individuals were used as controls. Incubation of mock-cultured RBCs with control or patient plasma at 37 or 41°C did not alter the EI profiles (Figure 2A). 9 of 12 plasma samples from malaria patients increased the rigidity of cultured rings compared to control plasma when incubation was performed at 41°C, but not at 37°C (Figure 2B–C; Supplementary Figure 2D–L). The median (interquartile range) of EI max values of cultured rings incubated with patient plasma was 0.52 (0.48 – 0.52) (for 37°C) and 0.46 (0.42 – 0.49) (for 41°C). The values at 41°C coincide with previous EImax estimates for 100% ring parasitemia (0.47, 0.46 – 0.48)^7^ (pink band on Figures 2B–C). This suggests either that patient plasma drastically alters *Pf*RBC deformability or that it alters uRBCs deformability as well. At the individual level, three patient plasma samples induced very low EI max values at 37°C and/or 41°C (Supplementary Figure 2J–L) that cannot be explained only by the contribution of ring infected RBCs. Confirming our interpretation of RBC deformability of patients at day0, these results suggest that the reduced EI values of cultured rings incubated with malaria plasma from day0 amalgamates the contribution of both uRBCs and ring-harboring RBCs. These data also indicate that the reduced deformability of uRBCs depended on the presence of parasites in the culture, since deformability of mock-cultured RBCs incubated with the malaria plasma at 37 or 41°C was not affected (Figure 2A).

There was no correlation between the plasma concentrations of L-arginine or nitrite and EI max values of cultured rings incubated at 37°C with patient plasma (Figure 2D–E). However, when incubation was done at 41°C, the EI max values of cultured rings were strongly and positively correlated with the plasma concentration of L-arginine (r = 0.89; p < 0.0001) (Figure 2F). There was no significant correlation with nitrite concentrations (r = 0.35; p = 0.12) (Figure 2G).

### L-arginine-dependent intra-parasite production of nitric oxide

**Figure 3:**
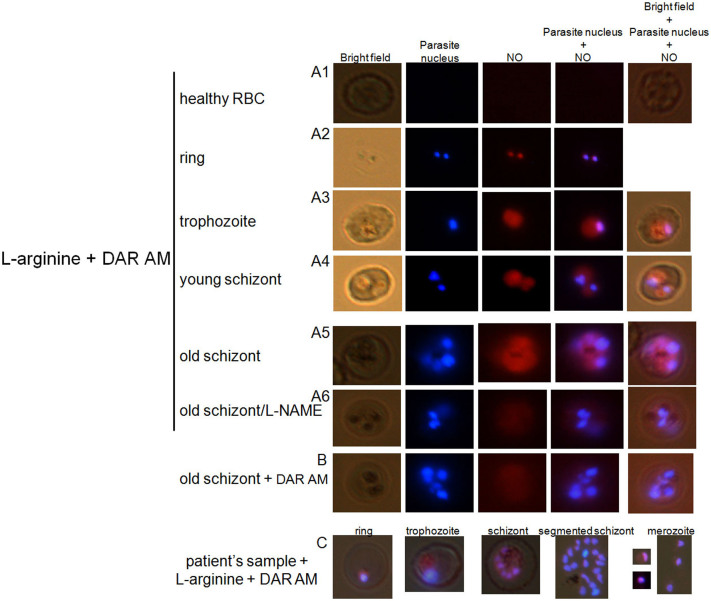
Evidence of L-arginine-dependent production of NO in *P. falciparum*-harboring RBCs. Uninfected RBCs and *Pf*RBCs incubated with the fluorescent dye 4,5-diaminofluorescein diacetate (DAR-4M AM, a specific NO probe) and L-arginine showed intense fluorescence in the parasite (red color), increasing with the developmental stage of the parasite, indicating intra-parasite production of NO (A1-5). Preincubation of *Pf*RBCs with 3 mM L-NAME, a competitive inhibitor of mammalian NOS, caused a significant reduced fluorescence (B). Intense intra-parasite fluorescence from a *P. falciparum* sample freshly isolated from malaria patients and incubated *in vitro* during 48 hours ((C), one representative sample).

The positive correlation between L-arginine and nitrite plasma levels in malaria patients (Figure 1G), suggested an L-arginine- and NOS-dependent production of NO. We therefore investigated whether NOS activity could be evidenced within uRBCs and *Pf*RBCs. First, we studied whether labeling with the fluorescent indicator 4,5-diaminofluorescein diacetate (DAR-4M AM)^32^ was influenced by L-arginine. Parasite nucleus was labeled in blue with Hoechst. We found that NO production (red color) increased with parasite maturation, and wasn't detectable by this method in healthy RBCs (Figure 3A1–5). Ring and mature *Pf*RBCs incubated with L-arginine showed intense fluorescence within the parasite (red color), but not in the host RBC cytoplasm or cytoplasmic membrane (Figure 3A1–5), indicating intra-parasite accumulation of NO. We were able to detect NO accumulation within both the cytoplasm and the nucleus of the parasite. *Pf*RBCs did not fluoresce in the absence of DAR-4M AM (not shown). Preincubation of *Pf*RBCs with 3 mM L-NAME, a competitive inhibitor of NOS, caused a significant reduction of the intra-parasite fluorescence (Figure A6). We found intense intra-parasite fluorescence at different parasite developmental stages when this assay was conducted on *P. falciparum* samples freshly isolated from malaria patients and incubated *in vitro* during 48 hours (representative example shown Figure 3C). Our data thus confirm previous evidence of intra-parasite production of NO^33^, but we attribute it to an arginine- and NOS-dependent pathway.

### L-arginine increases the deformability of healthy RBCs or cultured trophozoites *in vitro*

**Figure 4:**
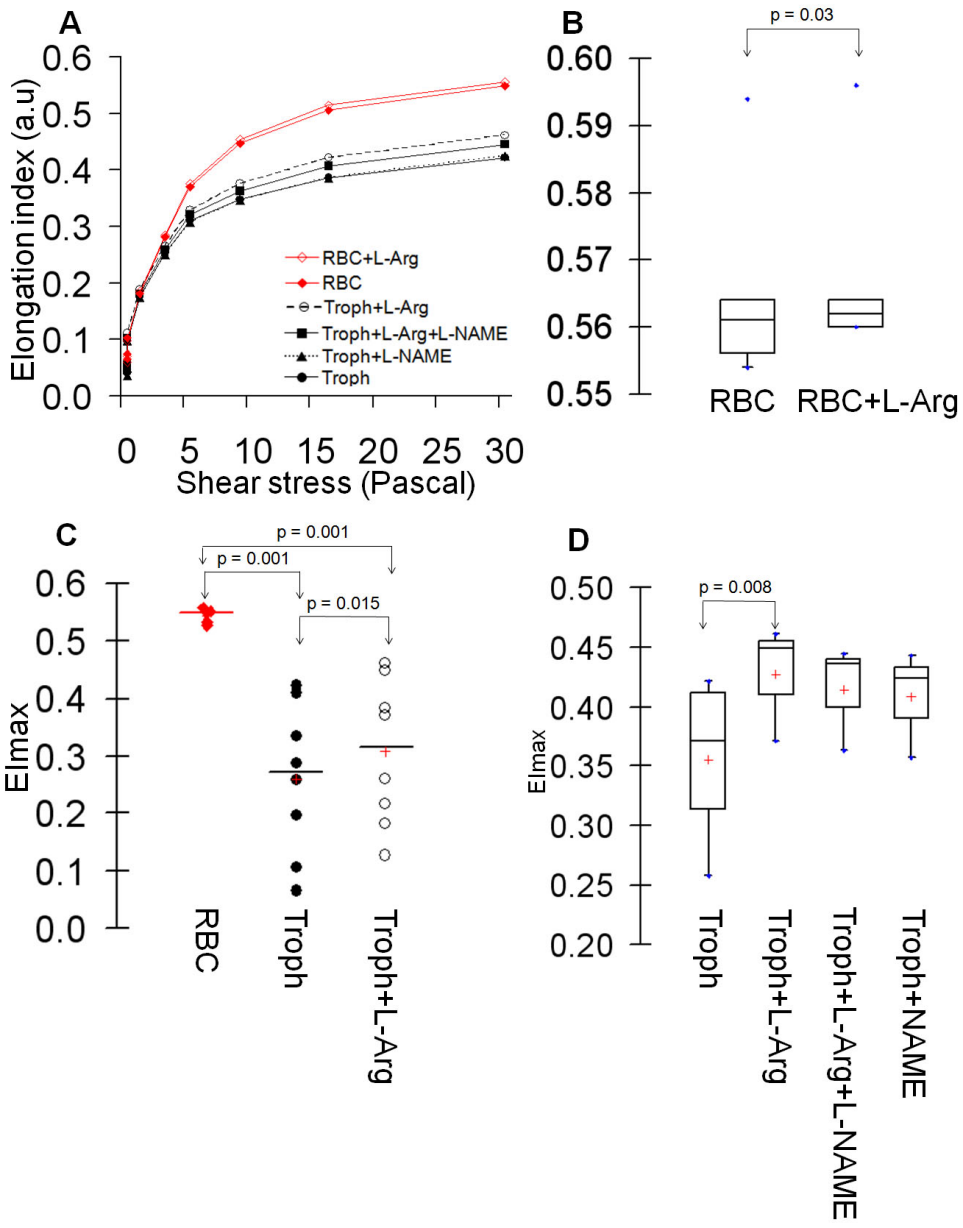
L-arginine induces increased deformability of healthy red blood cells or *P. falciparum* cultures. Deformability profile of healthy RBCs (RBC) or trophozoite culture (Troph) incubated with 4 mM of L-arginine (L-Arg) and/or 3 mM of L-NAME (A). EImax of healthy RBCs without (RBC) and with 4 mM of exogenous L-arginine (RBC + L-Arg) (B) or trophozoite culture incubated with 4 mM of L-arginine (C) and 3 mM L-NAME (D).

Since there was an arginine-dependent production of NO within *Pf*RBCs (Figure 3), we evaluated whether it could affect cellular deformability. We incubated healthy RBCs or trophozoite culture*s* in the presence of L-arginine and/or L-NAME. Trophozoite cultures were preferred over ring cultures because of their higher production of NO (Figure 3) and markedly reduced cellular deformability^7^,^9^. Upon addition of L-arginine, there was a slightly but reproducible increased deformability of cultured healthy RBCs or *P. falciparum* trophozoites (Figure 4A–C), a phenomenon partially inhibited by L-NAME (Figure 4D). This inhibition was not statistically significant. These results were consistent with the notion that L-arginine can modulate in an NO-dependent pathway the deformability of uRBCs and *Pf*RBCs *in vitro*.

### Presence of nitric oxide synthase within healthy or parasitized RBCs

**Figure 5:**
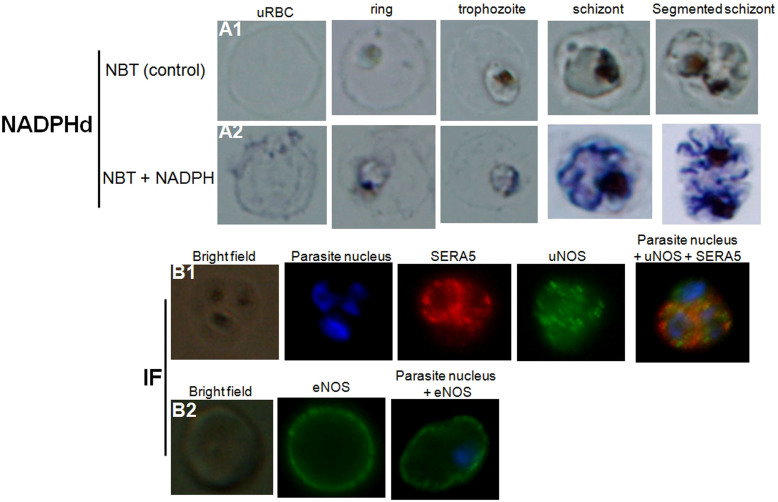
Detection of nitric oxide synthase in both healthy and *P. falciparum*-harboring red blood cells. NADPH-diaphorase activity within healthy RBCs or *Pf*RBCs at different stages of parasite development (A). The panel (A1) represents the negative control (uRBCs or *Pf*RBCs incubated with NBT without NADPH). NADPHd staining (blue color) was observed in both uRBC and parasite (A2). Identification by immunofluorescence assay (IF) of NOS within uRBCs or *Pf*RBCs using rabbit polyclonal antibody against uNOS (B1, green) or mouse monoclonal antibody against eNOS (B2, green). To differentiate infected from uninfected RBCs, parasite nucleus was labeled in blue with Hoechst 33342. A monoclonal antibody against parasite SERA5 was used to localize the parasitophorous vacuole (red).

We next investigated whether NOS could be detected in uRBCs and *Pf*RBCs. We used the convenient NADPH-diaphorase (NADPHd) staining to localize NOS in fixed cells^34^,^35^ (NOS is the only fixative-insensitive enzyme that can use NADPH to reduce a tetrazolium salt). Compared to the negative control (NBT without NADPH) (Figure 5A1), NADPHd staining (blue color) was detected in both uRBC and parasite (Figure 5A2). The most intense staining by the NBT formazan product was seen both in the cytoplasm and the nucleus at all developmental stages of the parasite (Figure 5A2).

It has been shown that the histochemical staining may not to be specific for NOS because, NADPHd activity can also be produced by other enzymes^34^,^36^. Therefore, to support the argument that NOS is present in *Pf*RBCs, a polyclonal antibody raised to a region conserved in the murine NOS isoforms was used for immunofluorescence assay (Figure 5B1). This antibody is designated universal NOS (uNOS) as it can detect all mammalian isoforms of NOS and has also been shown to detect NOS in *Drosophila*^37^. The uNOS antibody staining was localized only in the parasite, consistent with NADPHd labeling (Figure 5B1). This was clearly different from the staining produced by a mouse monoclonal antibody against human eNOS, which as reported^23^ labeled the RBC membrane of uRBCs and *Pf*RBCs (Figure 5B2). These data strongly suggested the existence of a *P. falciparum* NOS-like enzyme.

## Discussion

The present study aimed to evaluate the role of L-arginine-dependent production of NO on the RBC deformability during *P.* *falciparum* malaria. In travelers returning from endemic areas (i.e. patients with limited or no preexisting immunity to *P. falciparum*), deformability of the peripheral RBCs was reduced and plasma levels of L-arginine were low during the acute attack. L-arginine plasma concentrations had returned to basal levels four weeks later, as did RBC deformability. A similar reduction of cellular deformability could be induced upon incubation of *P. falciparum* ring-stage cultures with patients' plasma collected during the clinical attack. This effect was particularly marked when incubation was carried out at 41°C, *i.e*., a body temperature frequently observed in febrile malaria patients. Plasma-induced alterations of cellular deformability of *P. falciparum* cultures correlated with L-arginine concentrations. Taken together, these results suggest that the reduced deformability of RBCs during malaria attacks may be due to the lower plasma concentration of L-arginine. This hypothesis is substantiated by the enhanced deformability of healthy RBCs or cultured trophozoites upon incubation *in vitro* with L-arginine. This effect of L-arginine on RBC deformability was at least in part NOS-dependent, as L-NAME, a specific inhibitor of mammalian NOS consistently reduced the phenomenon.

The reduced bioavailability of L-arginine in malaria patients and *P. falciparum* cultures may stem from *s*everal factors. *In vitro*, this could result from the high parasite arginase activity^26^,^27^, which has been shown to be the major determinant of L-arginine depletion in *P. falciparum* cultures^26^. This is consistent with the observation that the reduced deformability of uRBCs *in vitro* depended upon the presence of parasites in the culture, since the deformability of mock-cultured RBCs incubated with the malaria plasma samples was not affected (Figure 2A). In *P. falciparum*-infected patients, hypoargininemia may be driven by a variety of mechanisms, including malaria parasite arginase^26^,^27^, increased plasma levels of arginase released during RBC lysis^25^, cytokine-induced activation of arginase of host cells such as macrophages/monocytes and endothelial cells^24^,^38^, inadequate L-arginine adsorption by enterocytes, or endogenous biosynthesis or recycling^39^,^40^. All these processes concur to limit the bioavailability of NO by removing the substrate necessary for its production. Moreover, the bioavailability of NO may also be limited by the plasma accumulation of asymmetrical dimethylarginine that inhibits NOS in *P. falciparum*-infected patients^41^. These results are thus consistent with the interpretation that impaired RBC deformability during malaria may be associated with a dysfunction in the NO pathway that reduces its production as infection proceeds by progressively depleting its metabolic precursor. Given that inhibition of NOS reduces RBC filterability, a phenomenon restored by NO donor^21^,^22^, we can hypothesize that NO-dependent maintenance of cellular deformability may be crucial for successful RBC traversal through the microvasculature and inter-endothelial slits of the spleen red pulp^7^,^8^,^9^. Further experimental studies are needed to support this hypothesis as the relationship of RBCs filterability to the rheologic *in vivo* circumstances is still debated^42^,^43^.

Although NO production by host cells, including monocytes/macrophages and endothelial cells likely contributes to the overall picture *in vivo*, our data suggest that parasite-produced NO might come into play as well. We show evidence suggesting the presence of NOS activity within the parasite and a substantial increase of the basal DAR-4M AM signal upon addition of exogenous L-arginine to the incubation medium. The L-arginine-dependent DAR-4M AM signal was intense in the parasite. We confirmed the presence of eNOS in the membrane of RBCs reported by others^23^, but could not obtain convincing evidence of RBC-membrane associated enzymatic activity predicted from the immunolocalization of the human eNOS. This might result from the high affinity of hemoglobin for NO acting as a sink for all NO produced^28^,^44^. In addition, NO might be produced by an alternative pathway in the parasite that involves putative cytochrome b5/cytochrome b5 reductase activities coordinated with heme in the food vacuole^33^,^45^.

The mechanisms by which NO affects RBC deformability remain to be determined. NO is hydrophobic and accumulates in lipid membranes, where autoxidation to nitrite occurs *in vivo*^46^. NO may activate cellular soluble guanylyl cyclase (sGC) to produce cGMP, promoting phosphorylation of spectrin and cytoskeleton components. It may also stimulate Na^+^K^+^ATPase and Ca^2+^ATPase that regulate intracellular Ca^2+^-flows^19^,^21^,^22^,^47^. These processes are involved in the regulation of RBC intracellular volume and cytoplasmic viscosity^19^,^21^,^22^,^46^,^47^, properties known to affect the deformability of mammalian RBCs^2^,^48^.

We found a weak correlation between plasma levels of nitrite and deformability of whole RBCs during malaria attacks or of *P. falciparum* ring-stage cultures. This might be linked to the fact that the quantification of plasma nitrite (without nitrate) measures a minority of endogenous NO, as most nitrite is oxidized *in vivo* to nitrate. Nitrite may not be a good surrogate marker of NO, because the plasma nitrite levels are known to be influenced by a variety of NOS-independent factors^49^. Other mechanisms than the L-arginine and NO pathway may also be involved in modifying RBC deformability. In particular, the high level of reticulocytes observed in patients at day0 (Table 1) may contribute to the overall reduced deformability, since young RBCs are less deformable than mature RBCs^50^. Obviously other factors (proteins, glycolipids, hydroxynonenal) released in the plasma by the parasite^13^,^14^,^15^,^51^ or host cells^52^ could modulate deformability of *Pf*RBCs and uRBCs in malaria patients.

Some methodological limitations must be taken into account in this study. Firstly, the fact that the contribution of RBC diseases known to affect RBC deformability, such as α,β- thalassemia^53^,^54^ and sickle cell disease^55^ frequent in endemic areas, cannot be excluded since they were not tested in our population. However, their contribution is unlikely since the magnitude of the reduction of the EI values at day 0 was low (Supplementary Figure 1) compared to about the marked decrease observed in these RBC disorders using a similar technique^53^,^54^. Moreover, the malaria patients recruited here were not anemic (Table 1). Secondly, the concentration of 4 mM of L-arginine used for *in vitro* experiments is supraphysiological (about 40-fold higher than normal plasma concentrations in humans). Therefore, the results are of uncertain physiological significance. Thirdly, the fact that the centrifugation and freezing of plasma occurred within 2 hours, may result in underestimating the plasma concentrations of L-arginine since about 10% of L-arginine might be loosed within half an hour after blood collection due to artefactual *ex vivo* metabolism^56^. However, it is unlikely that these shortcomings substantially altered the main conclusion of our study, as control plasmas were processed in the same way. We thus interpret our findings as indicating involvement of the L-arginine and NO pathway in the altered mechanical properties of RBCs during clinical *P. falciparm* malaria.

In conclusion, the results presented here suggest that hypoargininemia during *P.* *falciparum* malaria probably limits NO production and contributes to reduced deformability of RBCs. Strategies to increase L-arginine and/or NO production could improve outcome by restoring RBC deformability thereby facilitating their circulation through systemic capillaries and splenic slits. Drugs aimed at improving RBC deformability in *P.* *falciparum* malaria are a promising area for future research.

## Methods

### Patients and blood collection

Adult patients (age range: 25 to 61 y) with a *P. falciparum* monoinfection, parasitemia > 0.1%, no signs or symptoms of severe malaria, and providing written informed consent were included in this observational study approved by the Necker Hospital Investigational Review Board (Paris, France). Parasitemia was determined on Giemsa-stained smears as per routine procedure. Except for the collection of 25 ml of venous blood at days 0 and 28, the capture of clinical data, current laboratory parameters and RBC deformability measurements, the study did not induce any modification of medical care. Antimalarial therapy was administered immediately after blood sampling as per decision of the attending physician. Blood samples were centrifuged within less than 2 hours after collection, and plasma samples were aliquoted and stored at −80°C.

### Measurement of RBC deformability

RBC deformability was measured by ektacytometry using a laser-assisted optical rotational cell analyzer (LORCA; Mechatronics, Hoorn, The Netherlands) as previously described^7^,^9^. The unit of RBC deformability, namely the elongation index (EI), was defined as the ratio between the difference between the 2 axes of the ellipsoid diffraction pattern and the sum of these 2 axes. RBC deformability was assessed over a range of shear stresses (0.3–30 Pa).

### Quantification of plasma L-arginine and nitrite concentration

Plasma L-arginine concentration was measured by high performance liquid chromatography at the CERBA Laboratory (Paris, France) on a thawed plasma sample. Nitrite concentration was measured using the Griess reaction as described^57^. Nitrite concentration was used as a marker for NO production.

### *In vitro* incubation assay of malaria patient plasma with *P. falciparum* ring-stage cultures

*P. falciparum* FUP-CB - alias FCR3-58 was cultured as described^59^. Cultures were synchronized by multiple successive gel flotation and sorbitol (5%) treatments until the parasites reinvaded erythrocytes within a 4-hour time window. Synchronized ring-stage (<9 hours of age) cultures were incubated with patient plasma at 10% hematocrit and cell deformability was determined after 2 hours of incubation. RBCs from the same RBC donor were cultured in absence of parasite and used as control (mock-cultured RBCs).

### NADPH-diaphorase assay

NADPH-diaphorase (NADPHd) activity was assayed as described^60^. Briefly, culture medium was removed and the RBCs were fixed for 5 minutes with 4% paraformaldehyde and rinsed with PBS. NADPHd activity was revealed by incubating cells at 37°C for 45 minutes with 1 mL PBS supplemented with 0.5 mL NADPH (1.25 mg/mL), nitroblue tetrazolium (NBT, 100 mg/mL), 0.2% Triton X-100 in 0.1 mol/L phosphate buffer (pH 8.0). The cells were washed and left in PBS for microscopy. Images were acquired on a Zeiss Axiovert 200 M microscope, using an Axiocam HRc camera controlled by Zeiss Axiovision software (all from Carl Zeiss, Heidelberg, Germany).

### Immunofluorescence assay

The following reagents were used to detect the human RBC NOS enzyme and/or the intraparasitic NOS: a mouse monoclonal antibody against human eNOS (610296, BD Transduction Laboratories), a rabbit polyclonal antibody raised to a region conserved in the murine NOS isoforms (PA1-039, ABR Affinity BioReagents), designated universal NOS (uNOS)^37^. A mouse monoclonal antibody against parasite SERA5 was used to detect the parasitophorous vacuole (a kind gift from Dr. Jean Christophe Barale, Institut Pasteur, Paris). Alexa Fluor 488-conjugated goat anti-mouse affinity-purified IgG and/or Alexa Fluor 594-conjugated goat anti-rabbit affinity-purified IgG were used as secondary antibodies (Invitrogen). The immunofluorescence assay was performed as described^23^. Parasite nuclei were stained with Hoechst 33342 (diluted 1:1000; Invitrogen, Carlsbad, CA; LSR; BD Biosciences, San Jose, CA). Slides were mounted with Vectashield medium (Vector Laboratories, Burlingame, CA) for imaging.

### Detection of NO and NO-derived reactive nitrogen species using DAR-4M AM

This was performed as described^33^ with a slight modification. Briefly, synchronous *P. falciparum* cultures at different stage of development (ring, trophozoite and schizont) were incubated with PBS-albumax supplemented with L-arginine (3 mM) and 1 μM DAR-4M AM (Calbiochem, EMD Chemical) for 30 min at 37°C and then washed three times with PBS before microscopic observation.

### *In vitro* incubation assay of healthy RBCs or *P. falciparum*-infected RBCs with exogeneous L-arginine

This assay was performed as described^23^ with a slight modification. Briefly, healthy RBCs of trophozoite-infected RBCs were incubated with L-arginine (over a concentration range), and/or with L-NAME (3 mM) or buffer (PBS, 1% albumax II), and deformability of RBCs was assessed after 1 hour incubation.

### Statistical analysis

The Wilcoxon signed-rank test for matched pairs was used to compare continuous outcomes at different time points. The Mann Whitney U test or OneWay ANOVA with a Tukey posthoc analysis was used to compare continuous outcomes of different groups at the same time point. The correlation between different continuous measures was determined using the Spearman correlation coefficient. All p-values were two-sided, and p-values of less than 0.05 were considered statistically significant. All statistical analyses were performed with SPSS statistical software (PASW statistic version 18).

## Author Contributions

J.R. performed research and analyzed data; P.A.B. designed research, performed research, analyzed data and wrote the paper; L.C. analyzed data, contributed vital analytical tools; O.M.P. and G.M. designed research, analyzed data and wrote the paper; I.S. designed research, performed research, analyzed data and wrote the paper. All authors reviewed the manuscript.

## Supplementary Material

Supplementary Information NEW SI FILE

## Figures and Tables

**Table 1:** Biological characteristics of included *P.* *falciparum* malaria patients during acute attacks (day0) and after total recovery (day28)

	Day 0	Day 28	*p value*
Age (years)	48 (25–61)		
Gender (female/male)	6/13		
Body temperature (\C)	38.7 (37–40.4)		
Origin of the patients, n(%)			
Africa	16 (67)		
Europe	8 (33)		
Parasitemia (%)	5 (3–7)	0	
Red blood cells (/mm^3^) (×10^6^)	3.9 (3.6–4.4)	3.7 (3.5–4)	*0.06*
Hemoglobin (g/dL)	11.8 (10.2–13.3)	11.5 (10.8–12.2)	*0.46*
Hematocrit (%)	34.2 (31.1–39.2)	33.6 (32–36.7)	*0.60*
MCV (fL)	87.5 (84–92.3)	91.5 (84.8–96)	*0.009*
MCHC (g/dL)	33.4 (32.7–34.1)	33.5 (32.5–34.3)	*0.76*
MCH (pg)	28.9 (28.1–31.3)	30.7 (28.6–32.2)	*0.009*
Lymphocytes (/mm^3^) (×10^3^)	0.8 (0.5–1.4)	2.1 (1.3–2.4)	*0.007*
Monocytes (/mm^3^) (×10^3^)	0.2 (0.1–0.5)	0.4 (0.3–0.5)	*0.12*
Neutrophils (/mm^3^) (×10^3^)	4.2 (2.5–4.4)	2 (1.6–3)	*0.005*
Eosinophils (/mm^3^)	19 (7.5–33)	161.5 (119–303.3)	*< 0.0001*
Basophils (/mm^3^)	21 (12–45.5)	27.5 (15.3–63.8)	*0.65*
Platelets (/mm^3^) (×10^4^)	6 (3.6–10.2)	22.3 (17.7–25.4)	*0.0004*
Reticulocytes (/mm^3^) (×10^4^)	9.3 (6.3–19)	8.4 (7.5–10.7)	*0.007*
Total lgG (g/L)	8.2 (6.2–9.9)	11.5 (8.5–13.6)	*0.001*
Total lgM (g/L)	0.9 (0.7–1.6)	1.4 (0.9–1.9)	*0.09*
L-arginine (μM)	28 (20–33.5)	40.5 (30–50.5)	*0.004*
Nitrite (μM)	12.6 (6.1–24)	nd	

Values are median (interquartile range); n, number of patients; nd, not determined. The sex and origin were not recorded for 11 and 6 patients respectively.
