# Supplementary material for: Reduced erythrocyte deformability associated with hypoargininemia during Plasmodium falciparum malaria
Source: Sci Rep. 2014 Jan 20;4:3767. doi: 10.1038/srep03767 (PMC3895919; doi:10.1038/srep03767)

Supplementary information for:

Manuscript title **"Reduced erythrocyte deformability associated with hypoargininemia during *Plasmodium falciparum* malaria"** from Julianna Rey[1,2], Pierre A Buffet[3,4], Liliane Ciceron[3,4], Geneviève Milon[5], Odile Mercereau-Puijalon[1,2], Innocent Safeukui[1,2,6*]

1 Institut Pasteur, Immunologie Moléculaire des Parasites, Département de Parasitologie Mycologie, F- 75015 Paris, France

2 CNRS, URA2581, F- 75015 Paris, France

3 INSERM - UPMC (Paris 6 University) UMRs945, F-75013 Paris, France

4 AP-HP, Department of Parasitology, Pitié Salpétrière Hospital, F-75013 Paris, France

5 Institut Pasteur, Immunophysiologie et Parasitisme, Département de Parasitologie Mycologie, F-75015 Paris, France

6 Center for Rare and Neglected Diseases, and Department of Biological Sciences, University of Notre Dame, Notre Dame, Indiana, USA

*Corresponding author new mailing address: Innocent Safeukui, University of Notre Dame, Department of Biological Sciences, Notre Dame, IN 46556, United States of America, Phone 574-631-3227; Fax 574-631-1648, E-mail: innocent.safeukui@nd.edu

Supplementary figure 1

Deformability profile of whole RBCs obtained from 30 *P. falciparum* malaria patients at day0 compared to healthy controls (**A-C**). In panels A, B and C, the healthy controls are presented as the mean of EI ± standard deviation of the means of blood samples from 30 healthy subjects who have never travelled to malaria endemic areas.

Supplementary figure 2

Reduced deformability of synchronous *P. falciparum* ring cultures upon incubation with malaria patients' plasma from day0. Deformability profiles of ring cultures incubated at 37 or 41°C with malaria patient's plasma collected at day0 or with control plasma (**A-L**). The same symbols were used for all panels, indicated in panel **A.** The parasite density of ring cultures used for the assay is indicated for each patient on top of the respective graph.

Supplementary Figure 1

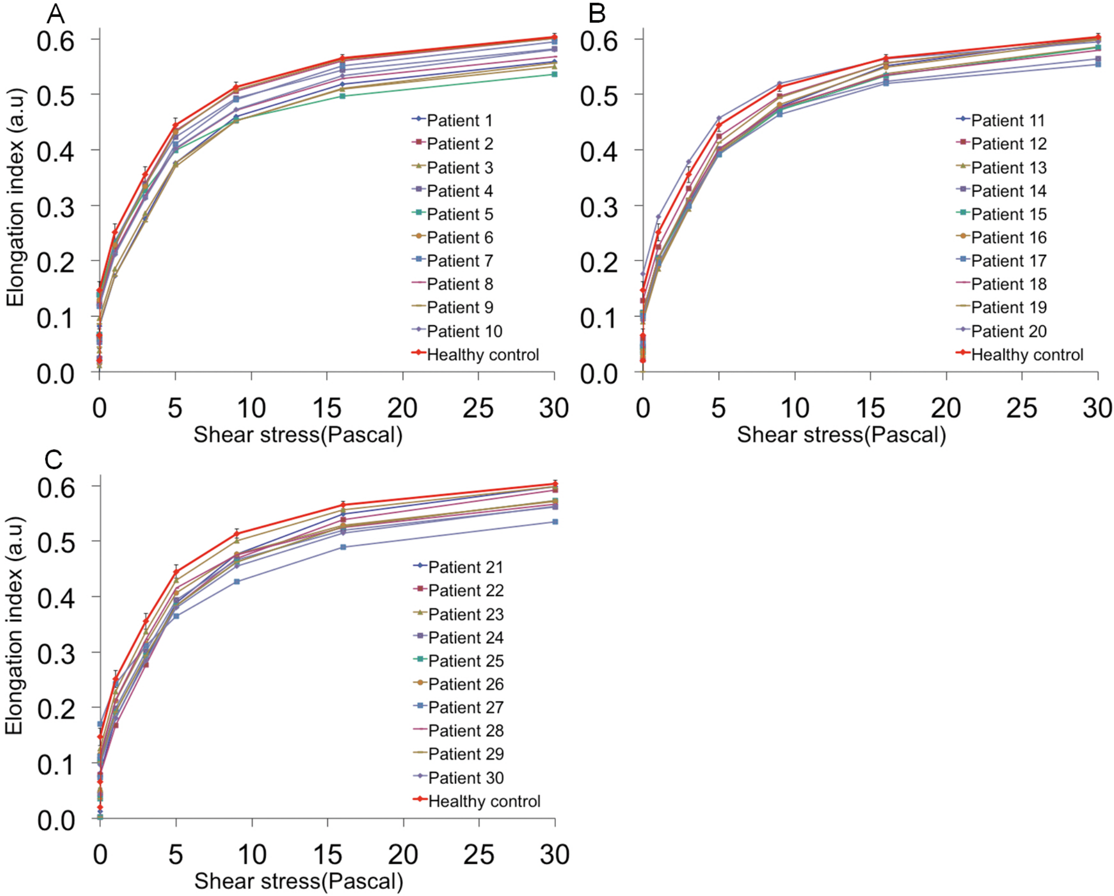

Supplementary Figure 2

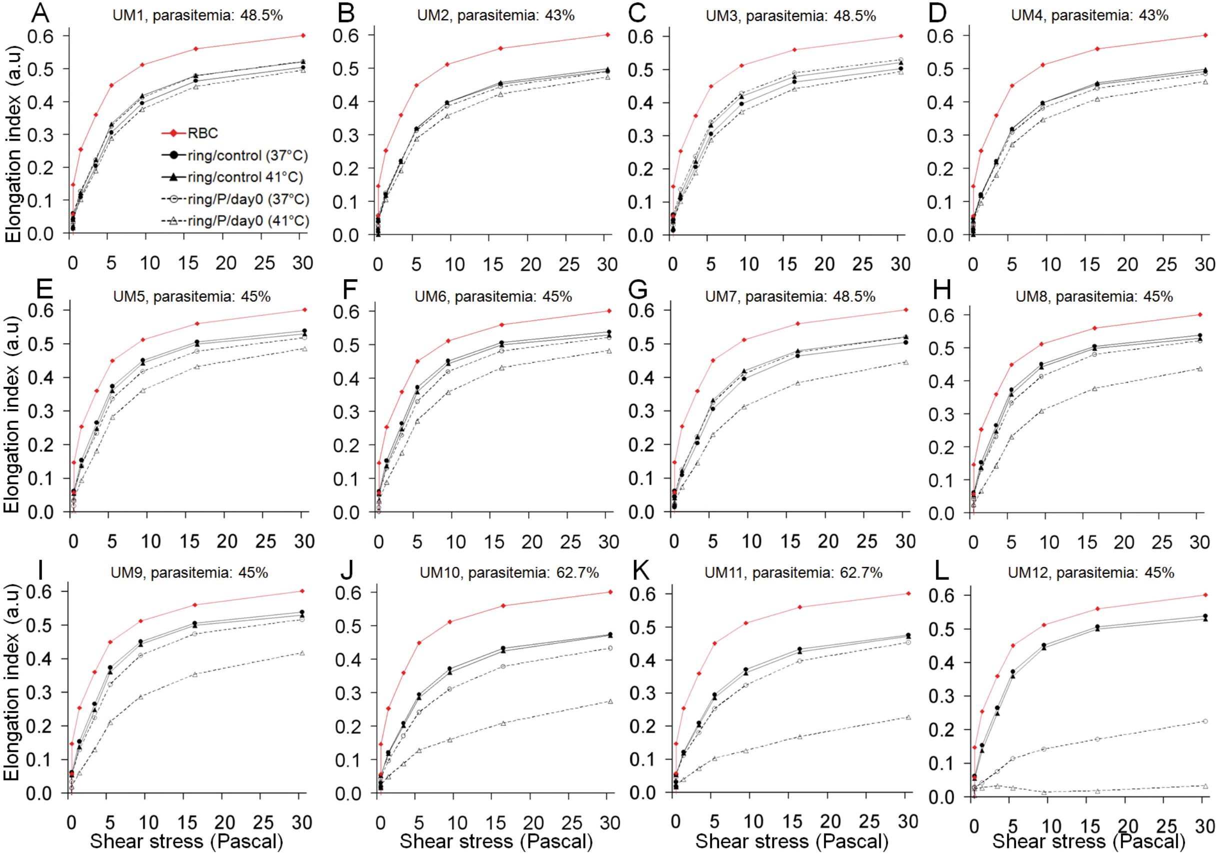

Supplement: Supplementary Information — NEW SI FILE [file srep03767-s1.pdf]
